# DNA plasmid vaccine carrying *Chlamydia trachomatis (Ct)* major outer membrane and human papillomavirus 16L2 proteins for anti-*Ct* infection

**DOI:** 10.18632/oncotarget.16601

**Published:** 2017-03-27

**Authors:** Ledan Wang, Yiqi Cai, Yirong Xiong, Wangqi Du, Danwei Cen, Chanqiong Zhang, Yiling Song, Shanli Zhu, Xiangyang Xue, Lifang Zhang

**Affiliations:** ^1^ Department of Gynecology, The Second Affiliated Hospital of Wenzhou Medical University, Wenzhou 325035, Zhejiang, China; ^2^ Department of Microbiology and Immunology, Institute of Molecular Virology and Immunology, Wenzhou Medical University, Wenzhou 325035, Zhejiang, China; ^3^ Department of Gastrointestinal, The First Affiliated Hospital of Wenzhou Medical University, Wenzhou 325035, Zhejiang, China

**Keywords:** Chlamydia trachomatis, major outer membrane protein, multi-epitopes, human papillomavirus, vaccine

## Abstract

*Chlamydia trachomatis* (*Ct*) is one of the most frequently encountered sexual infection all over the world, yielding tremendous reproductive problems (e.g. infertility and ectopic pregnancy) in the women. This work described the design of a plasmid vaccine that protect mice from *Ct* infection, and reduce productive tract damage by generating effective antibody and cytotoxic T cell immunity. The vaccine, s was composed of MOMP multi-epitope and HPV16L2 genes carried in pcDNA plasmid (i.e. pcDNA3.1/MOMP/HPV16L). In transfection, the vaccine expressed the chimeric genes (i.e. MOMP and HPV16L2), as demonstrated via western blot, RT-PCR and fluorescence imaging. *In vitro*, the vaccine transfected COS-7 cells and expressed the proteins corresponding to the genes carried in the vaccine. Through intramuscular immunization in BALB/c mice, the vaccine induced higher levels of anti-*Ct* IgG titer, anti-HPV16L2 IgG titer in serum and IgA titer in local mucosal secretions, compared to plasmid vaccines that carry only *Ct* MOMP multi-epitope or HPV16L2 chimeric component only. In mice intravaginally challenged with *Ct*, the vaccines pcDNA3.1/MOMP/HPV16L2 generated a higher level of genital protection compared to other vaccine formulations. Additionally, histochemical staining indicated that pcDNA3.1/MOMP/HPV16L2 eliminated mouse genital tract tissue pathologies induced by *Ct* infection. This work demonstrated that pcDNA/MOMP/HPV16L2 vaccine can protect against *Ct* infection by regulating antibody production, cytotoxic T cell killing functions and reducing pathological damage in mice genital tract. This work can potentially offer us a new vaccine platform against *Ct* infection.

## INTRODUCTION

*Chlamydia trachomatis* (*Ct*) is one of the primary causes in reproductive tract infections worldwide [1]. Globally, *Ct* causes more than 92 million sexually transmitted infections, yielding long term reproductive problems (e.g. infertility and ectopic pregnancy) in the women patients. Clinically, *Ct* is regarded as an immunologically sensitizing infection, and human immunity is unable to resist *Ct* completely due to the complicated evasion mechanisms of *Ct*. Additionally, *Ct* usually replicates within the epithelial cells, preventing itself from the immune effectors by using a sanctuary shield [2]. Beyond this, *Ct* also employs multiple other strategies to escape human immune systems, such as avoiding the antibody detection by presenting antigenically diverse surface proteins, inducing the apoptosis of activated effector T cells against the infection, etc. [3]. Thus, theses tricks in *Ct* pathobiology require us to design novel effective *Ct* vaccines by outdoing the immunological and biological barriers [4].

Existing dramatic challenges in chlamydial vaccine design include selecting appropriate candidate antigens and developing effective delivery vehicles. Of note is that the major outer membrane proteins (MOMP) account for over 60% of the *Ct* membranes. Thus MOMP are viewed as a major target against *Ct* infection [5, 6]. However, MOMP are generally weak immunogens and generate insufficient immunity against *Ct*, limiting the potential protective effects of the vaccines. Human papillomavirus 16L2 (HPV16L2) protein can help us address this issue by enhancing the immunogenicity of multi-epitope peptides [7]. HPV16L2 is an innocuous and highly antigenic protein that is composed of several common neutralization epitopes, with cross-reactivity against diverse HPV types [8, 9]. During immunization, antibodies against HPV16L2 peptides cross-react with cutaneous and mucosal HPV types, generating potent innate and adaptive immunity that can be harnessed to combat other infections including *Ct* [8, 10]. Importantly, studies indicate *Ct* may act as a cofactor that yields HPV infection in the genital tract [11–13], and no effective vaccine against *Ct* or against a broad spectrum of HPV infections is licensed currently. Thus, vaccines comprised of *Ct* and *HPV* components could be used to protect human against both *Ct* and HPV infections. In this study, we constructed a recombinant plasmid vaccine that can express *Chlamydial* MOMP multi-epitopes and HPV16L2, and employed this chimeric vaccine against *Chlamydia trachomatis* infections by generating antibody-specific and cytotoxic T cell immunity. In mice infected with *Ct*, the vaccine can help clear the infections effectively and eliminate the genital tract tissue damage induced by the infection.

## RESULTS

### pcDNA3.1(+)/MOMP/HPV16L2 plasmid was designed and constructed

**Figure 1:**
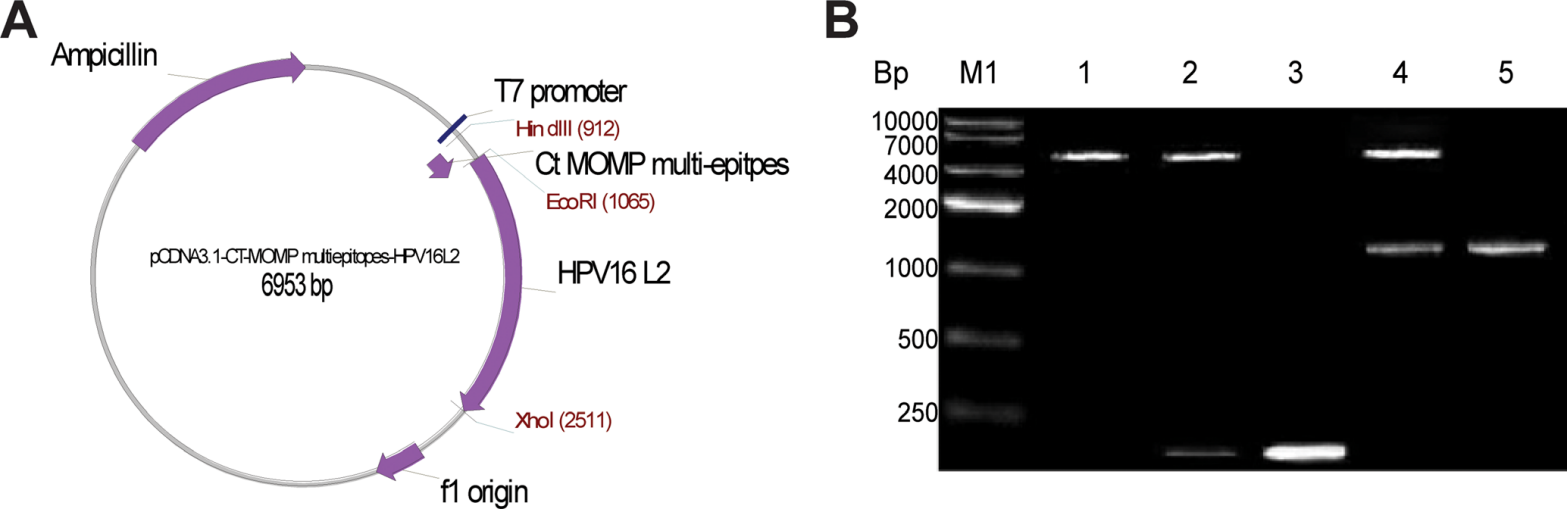
Schematic picture showing pcDNA3.1/MOMP/HPV16L2 plasmid structure and analysis of the plasmid components via enzyme digestion and PCR amplification (**A**) Schematic picture illustrating the structure of chimeric plasmid containing MOMP/HPV16L2. The plasmid is 6953 bp in length, and contains an ampicillin resistant section that helps the plasmid to survive in ampicillin-containing medium and selects specifically for the cells containing the plasmid. It also contains a T7 promotor, HPV16 L2 and f1 origin sections. (**B**) Confirming the construction of pcDNA3.1/MOMP/HPV16L2 via digestion and PCR amplification. Upon digestion of pcDNA3.1/Ct MOMP/ HPV16L2 with HindIII + EcoRI, there was an extra visible band at the length of 159 bp band (Lane 1), compared to the sample with no digestion (Lane 2). Amplification of *Ct* MOMP multi-epitope showed a band at 159 bp band, confirming the existence of *Ct* MOMP multi-epitope in the plasmid (Lane 3). After digestion of pcDNA3.1/MOMP/HPV16L2 with EcoRI + XhoI, an extra visible band at 1456 bp position was observed (Lane 4). Amplification of HPV16L2 products through PCR showed a band at the same position (i.e. 1456 bp, Lane 5), confirming the addition of HPV16L2 components in the plasmids.

Figure 1A illustrated the structure of the plasmid vaccine (pcDNA3.1/MOMP/HPV16L2) designed for this study. Briefly, the plasmid vaccine is 6953 bp in length, and contains two therapeutic genes: *Ct* MOMP multi-epitope and HPV16L2. Other major parts of the plasmid also include: a promotor (i.e. T7), HPV16 L2 and f1 origin sections (Figure 1A). To assess the successful construction of pcDNA3.1/MOMP/HPV16L2, the plasmid was digested with restriction enzymes (i.e. *Xho*I, *Hind*III, and *EcoR*I). With the condition of no restriction enzymes digestion, only one visible band between 4000 to 7000 bp was observed under RT-PCR analysis (Figure 1B Lane 1). Upon the digestion with HindIII and EcoRI enzymes, there was an extra visible band, with a length around 159 Bp. This band fits in the length of *Ct* MOMP multi-epitope, indicating the incorporation of the multi-epitope into the plasmid (Figure 1B Lane 2). PCR amplification of *Ct* MOMP multi-epitope illustrated a more vivid band at around 159 bp (Figure 1B Lane 3). Thus, the band in lane 2 and lane 3 together confirmed the incorporation of *Ct* MOMP multi-epitope into the plasmid. Similarly, upon digesting pcDNA3.1/MOMP/HPV16L2 with EcoRI and XhoI, we observed a band at 1456 bp position, which is the size HPV16L2 product, indicating HPV16L2 gene was incorporated into the plasmid (Figure 1B Lane 4). PCR amplification of HPV16L2 illustrated the band at 1456 bp (Figure 1B Lane 5). This band, together the band in Lane 4, confirms the incorporation of HPV16L2 gene into the plasmid. Overall, the PCR study demonstrated that both *Ct* MOMP and HPV16L2 genes were incorporated into the plasmid.

### DNA sequencing illustrated that HPV16L2 gene was successfully constructed into pcDNA3.1(+) plasmid

**Figure 2:**
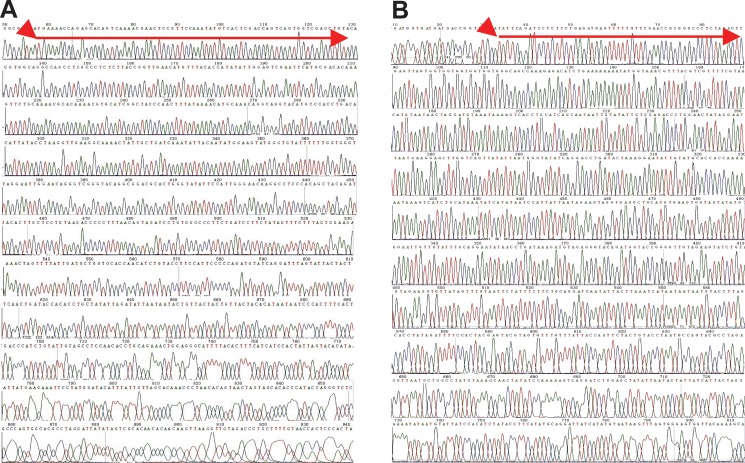
(**A**) The orientation sequence of recombinant plasmid pcDNA3.1/MOMP/HPV16L2. (**B**) The reversed sequence of recombinant plasmid pcDNA3.1/MOMP/HPV16L2.

In this study, while the pcDNA3.1 plasmid serves as a carrier, HPV16L2 gene plays significant role in enhancing the immunogenicity of pcDNA3.1/MOMP/HPV16L2 plasmid. Thus, using gene sequencing techniques, we identified the successful addition of HPV16L2 into the plasmid. Briefly, according to the gene sequence from China National Gene Sequence Bank (No. NC_001526), we designed HPV16L2 gene. The two components, pcDNA3.1 and HPV16L2, were then treated with restriction enzymes, EcoRI and XhoI, respectively, followed by reconstruction into pcDNA3.1/HPV16L2. We next used T7 as promoter primer and BGH as reverse primer for forward and reverse sequencing, respectively. Both the forward and reverse sequencing illustrated that the inserted HPV16L2 gene had the same sequence to the design, demonstrating the successful design of pcDNA3.1/HPV16L2 plasmid (Figure 2A and 2B).

### *Ct* MOMP and HPV16L2 mRNAs was successfully amplified in COS-7 cells

**Figure 3:**
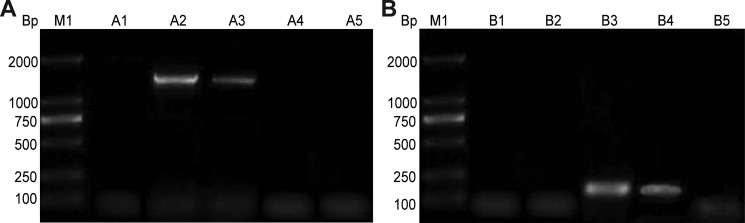
Identification of HPV16L2 and Ct MOMP multi-epitope gene expression in transfected COS-7 cells (**A**) Assessing the expression of HPV16L2 gene in COS-7 cells. Lane A1: COS-7 cells transfected with pcDNA3.1. Lane A2: Cells transfected with pcDNA3.1/MOMP/HPV16L2. LA3: cells transfected with pcDNA3.1/HPV16L2. Lane A4: Cells transfected with pcDNA3.1/MOMP. Lane A5: COS-7 cells with no transfection. (**B**) Measuring the expression of Ct MOMP multi-epitope gene in COS-7 cells. Lane B1: COS-7 cells transfected with pcDNA3.1. Lane A2: Cells transfected with pcDNA3.1/HPV16L2. LB3: Cells transfected with pcDNA3.1/MOMP/HPV16L2. Lane B4: Cells transfected with pcDNA3.1/MOMP. Lane B5: COS-7 cells with no transfection. The measurement was performed on the same gel, and the image was cropped into two Figures to make the description more clear.

**Figure 4:**
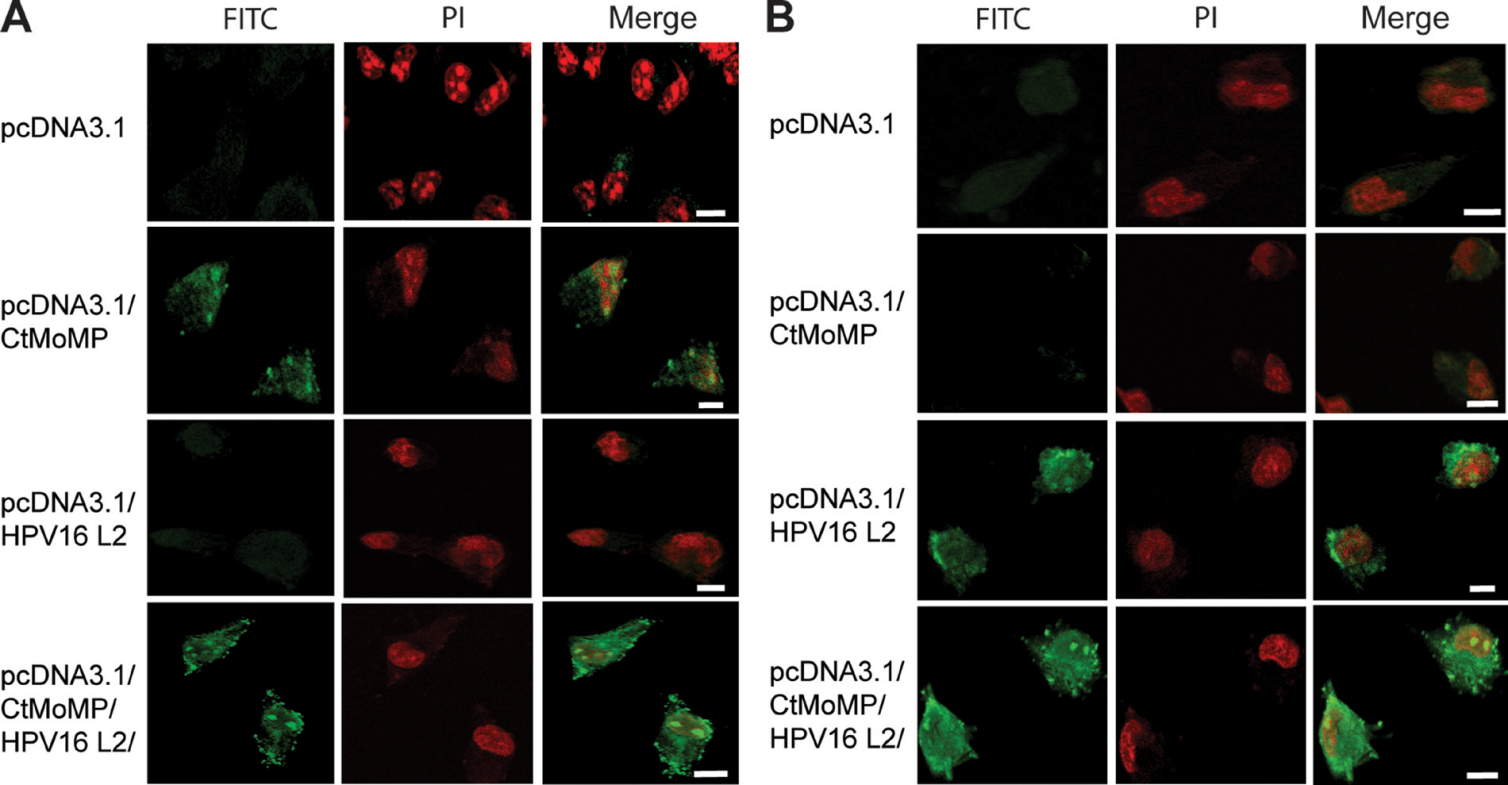
Confocal microscope imaging confirmed the expression of Ct MOMP and HPV16L2 in COS-7 cells after transfection The cells were transfected with different types of plasmids for 2 days, and then stained (**A**) anti-Ct-FITC polyclonal antibody, or (**B**) anti-HPV16L2-FITC polyclonal antibody. In both groups of tests, cells were transfected with pcDNA3.1, pcDNA3.1/MOMP, pcDNA3.1/HPV16L2 or pcDNA3.1/MOMP/HPV16L2. Transfection of cells with plasmids that contain Ct MOMP multi-epitope showed the expression of Ct MOMP. Similarly, plasmids containing HPV16L2 yielded the expression of corresponding proteins in the cells. In both tests, nuclei was stained with PI (i.e. red color). Overall, the tests demonstrated that both Ct MOMP and HPV16L2 were functional after transfection. Scale bars are 10 μm.

We next tested the amplification of *Ct* MOMP and HPV16L2 mRNA in COS-7 cells. For the expression of HPV16L2, cells transfected with pcDNA3.1/MOMP/HPV16L2 or pcDNA3.1/HPV16L2 illustrated a band at 1456 bp (Figure 3, Lanes A2 and A3); instead, cells transfected with pcDNA3.1 or pcDNA3.1/MOMP plasmid did not show this band associated with HPV16L2 (Figure 3, Lanes A1 and A4, respectively). A similar method was used to confirm the expression of *Ct* MOMP component in COS-7 cells (Figure 4B). In cells transfected with pcDNA3.1/MOMP/HPV16L2 and pcDNA3.1/MOMP plasmids, there was a fragment visible at 159 bp, which was the expression from *Ct* MOMP (Figure 3, Lanes B3 and B4), indicating the successful amplification of *Ct* MOMP in the cells. As a contrast, cells transfected with pcDNA3.1 or pcDNA3.1/HPV16L2 plasmid did not show this band since these plasmids did not include *Ct* MOMP genes. Together, the tests in Figure 3 illustrated the successful expression of *Ct* MOMP and HPV16L2 mRNAs in COS-7 cells after transfection.

### Laser confocal microscopy analysis confirmed the recombinant protein expression from pcDNA3.1(+)/MOMP/HPV16L2 plasmid transfection

The expression of *Ct* MOMP multi-epitope or HPV16L2 epitopes in COS-7 cells was also analyzed using antibodies staining and microscopy imaging. Briefly, the HPV16L2 or *Ct* MOMP protein expression within the cells was fluorescence-labeled anti-HPV16L2 or anti-*Ct* antibody, and observed under fluorescence microscopy, as illustrated in green color. The cell nuclei was stained with PI, showing red color under the microscope (Figure 4A and 4B). Figure 4A showed the expression of *Ct* polyclonal protein in cells transfected with different types of plasmids. In cells transfected with pcDNA3.1, no HPV16L2 protein (i.e. green color) was observed (Figure 4A, row 1). Cells transfected with pcDNA3.1/MOMP showed a decent level expression of *Ct* protein (Figure 4A, row 2); as a contrast, cells transfected with pcDNA3.1/HPV16L2 indicated no *Ct* protein expression since there was no *Ct* MOMP component in the plasmid (Figure 4A, row 3). The use of pcDNA3.1/MOMP/HPV16L2 yielded a significant level of *Ct* protein expression, indicating the effective functionality of the plasmid vaccine (Figure 4A, row 4). Similarly, antibody staining and fluorescence imaging confirmed the successful expression HPV16L2 in cells transfected with pcDNA3.1/MOMP/HPV16L2. Briefly, using anti-HPV16L2 polyclonal antibody staining, we found no expression HPV16L2 protein in cells transfected with pcDNA3.1 or pcDNA3.1/MOMP (Figure 4B, row 1 and 2). As a contrast, cells transfected pcDNA3.1/HPV16L2 or pcDNA3.1/MOMP/HPV16L2 showed a significant level of HPV16L2 protein expression, respectively, indicating HPV16L2 was functional during transfection (Figure 4B, row 3 and 4). Additionally, using fluorescence microscopy, our study indicated that 80% of the cells are transfected, where the calculation is based dividing the number of transfected cells by the total number of cells. Together, using antibody staining and microscope imaging, the study revealed the successful expression of both Ct-MOMP and HPV16L2 genes after the transfection in COS-7 cells.

### Western blot analysis illustrated the expression of *Ct* and HPV16L2 proteins in COS-7 cells treated with pcDNA3.1/MOMP/HPV16L2 plasmid vaccine

**Figure 5:**
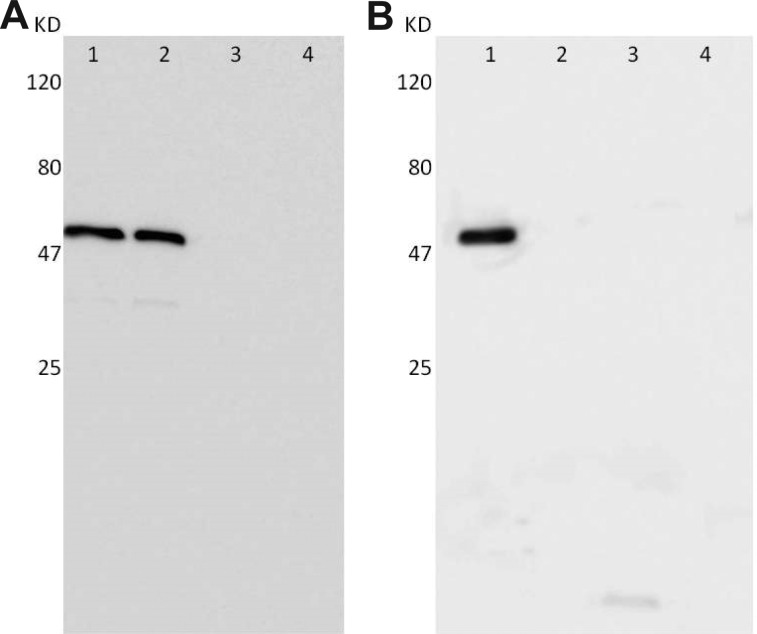
Western blot analysis showing the expression of *Ct* and HPV16L2 proteins in in COS-7 cells with the transfections Four group of plasmids were used for transfections: Sample 1: pcDNA3.1/MOMP/HPV16L2; Sample 2: pcDNA3.1/HPV16L2; Sample 3: pcDNA3.1/*Ct* MOMP multi-epitope; Sample 4: pcDNA3.1. (**A**) Expression of *Ct* MOMP/HPV16L2 or HPV16L2 protein in COS-7 cells, as tested by anti-HPV16L2 antibody. (**B**) Expression of *Ct* MOMP multi-epitope/HPV16L2 or *Ct* proteins, as tested by anti-*Ct* antibody staining.

In addition to confocal microscope imaging, the expression of *Ct* and HPV16L2 protein in COS-7 cells were assessed with Western blot as well. The fusion protein, *Ct* MOMP/HPV16L2, was around 60 kDa in size. Alternatively, *Ct* MOMP/HPV16L2 can also express as *Ct* and HPV16L2 proteins separately as well: the HPV16L2 protein has a size around 53 kDa and the *Ct* MOMP multi-epitope protein is 7 kDa. Western blot results confirmed the chimeric plasmid expression in cells engineered with pcDNA3.1/MOMP/HPV16L2 plasmid. Anti-HPV16L2 antibody staining during Western blot assessment confirmed the expression of HPV16L2 in cells transfected with plasmids containing HPV16L2 components (i.e. pcDNA3.1/MOMP/HPV16L2 and pcDNA3.1/HPV16L2), as illustrated in the band near 60 kDa (Figure 5A, sample 1 and sample 2). Antibody staining of the samples transfected with pcDNA3.1/MOMP did not show any visible bands (Figure 5A, sample 3). Similarly, anti-*Ct* antibody staining of samples from cells transfected with pcDNA3.1/MOMP/HPV16L2 plasmid revealed a visible band, indicating the expression of *Ct* in cells transfected with pcDNA3.1/MOMP/HPV16L2 (Figure 5B, sample 1). Cells transfected with pcDNA3.1/MOMP plasmid showed a slightly visible band at 7 kDa, indicating the expression of *Ct* protein (Figure 5B, sample 3). Overall, Western blot tests confirmed that both HPV16L2 and *Ct* proteins can be expressed in cells with pcDNA3.1/MOMP/HPV16L2 transfections.

### Chimeric vaccine boosted antibody-specific immune response

**Figure 6:**
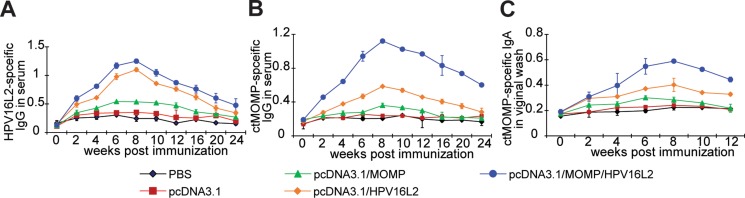
ELISA tests showing the levels of antibodies in mice receiving different plasmid immunizations In all tests, 5 groups of vaccines were selected: pcDNA3.1, pcDNA3.1/Ct MOMP, pcDNA3.1/HPV16L2, pcDNA3.1/Ct MOMP/HPV16L2. Mice received PBS treatments were employed as a control. (**A**) Levels of HPV16L2-specific serum IgG in mice receiving different immunization. (**B**) Levels of *Ct* MOMP specific IgG in serum. (**C**): Levels of Ct MOMP-specific secretory IgA in genital washes. *p < 0.05 vs. PBS group.

After confirming the functional expression of *Ct* and HPV16L2 proteins in cells with pcDNA3.1/MOMP/HPV16L2 transfection, we tested the antibody immunity generated by the plasmid vaccination in mice. Since the IgG, IgA in blood and vaginal fluids can protect mice from *Ct* infection, their levels in the immunized mice were analyzed by ELISA. Compared to the mice immunized with PBS, pcDNA3.1 or pcDNA3.1/MOMP, mice vaccinated with pcDNA3.1/MOMP/HPV16L2 or pcDNA3.1/HPV16L2 yielded significantly higher level of HPV16L2-specific IgG in mice (Figure 6A). Between weeks 2 and 24, the HPV16L2-specific IgG level in mice immunized with pcDNA3.1/MOMP/HPV16L2 was significantly higher than mice received pcDNA3.1 treatment or mice treated with pcDNA3.1/MOMP (*p* < 0.05). HPV16L2-specific IgG level in mice immunized with pcDNA3.1/MOMP/HPV16L2 was higher than mice treated with pcDNA3.1/HPV16L2 (Figure 6A). Similarly, a significantly elevated level of *Ct* MOMP-specific IgG was observed in mice vaccinated with pcDNA3.1/MOMP/HPV16L2 compared with the PBS group and mice immunized with pcDNA3.1/MOMP group (*p* < 0.05) (Figure 6B). A same trend was observed in the level of the *Ct*-specific IgA in the virginal wash: mice immunized with pcDNA3.1/MOMP/HPV16L2 had a higher level of *Ct*-specific IgA compare to the PBS-treated mice and mice vaccinated with pcDNA3.1/MOMP (*p* < 0.05) (Figure 6C). In all tests, the antibody levels started to increase upon immunization, reaching a peak at week 8 and gradually returning to a normal level in the following weeks (Figure 6A – 6C).

### pcDNA3.1/MOMP/HPV16L2 plasmid vaccine triggered *Ct*-specific cytotoxic T-lymphocyte (CTL) responses

**Figure 7:**
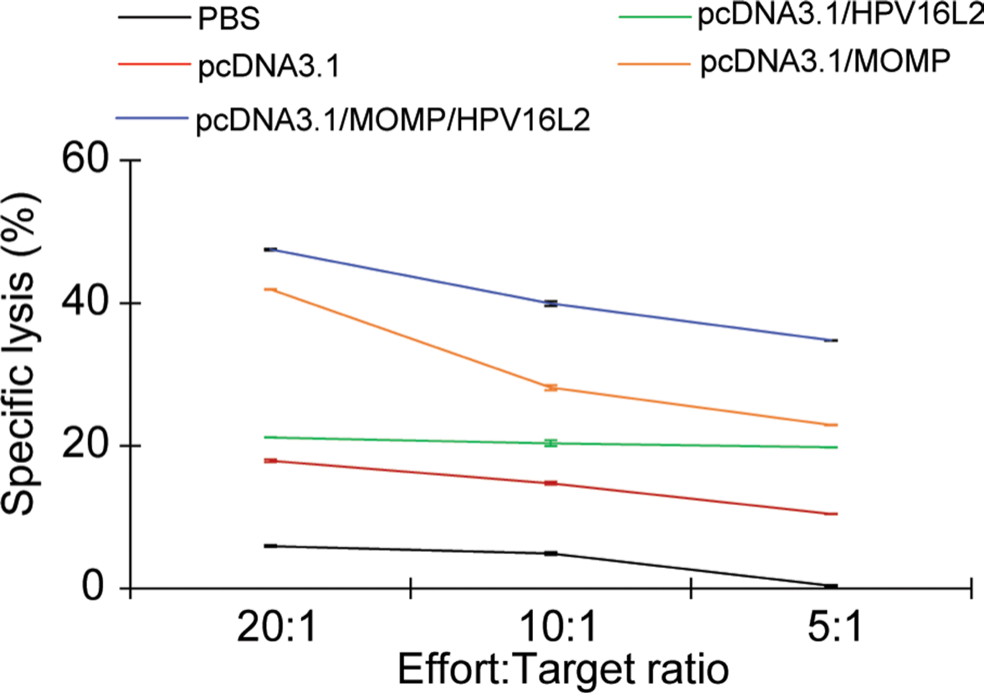
Cytotoxic T-lymphocyte (CTL) assay measuring the Ct-specific T cell immunity T cells from mice treated with pcDNA3.1/ MOMP/HPV16L2 had a more potent cytotoxic Ct-specific T cell lysis potency. pcDNA3.1/MOMP generated the second highest potent T cell immunity among the groups. No significant difference were observed among the pcDNA 3.1, pcDNA3.1/HPV16L2, and the PBS group. *p* < 0.05 between pcDNA3.1/MOMP/HPV16L2 and PBS group.

In this study, CTL assay was used to assess the *Ct*-specific T cell immunity. A higher level of *Ct*-specific cytotoxic activity was observed in mice immunized with pcDNA3.1/MOMP/HPV16L2, compared to mice treated with PBS or pcDNA3.1 plasmid (*p* < 0.05) (Figure 7). T cells from mice vaccinated with pcDNA3.1/MOMP/HPV16L2 were more cytotoxic than those T cells from mice receiving pcDNA3.1/MOMP irrespective of E/T ratio (*p* < 0.05). No significant differences existed between the pcDNA3.1/HPV16L2 and other control group (i.e. mice treated with PBS, mice immunized with pcDNA3.1 plasmid (Figure 7).

### pcDNA3.1/MOMP/HPV16L2 plasmid vaccine induced protective immunity in mice challenged with *Ct* intra-vaginally

**Figure 8:**
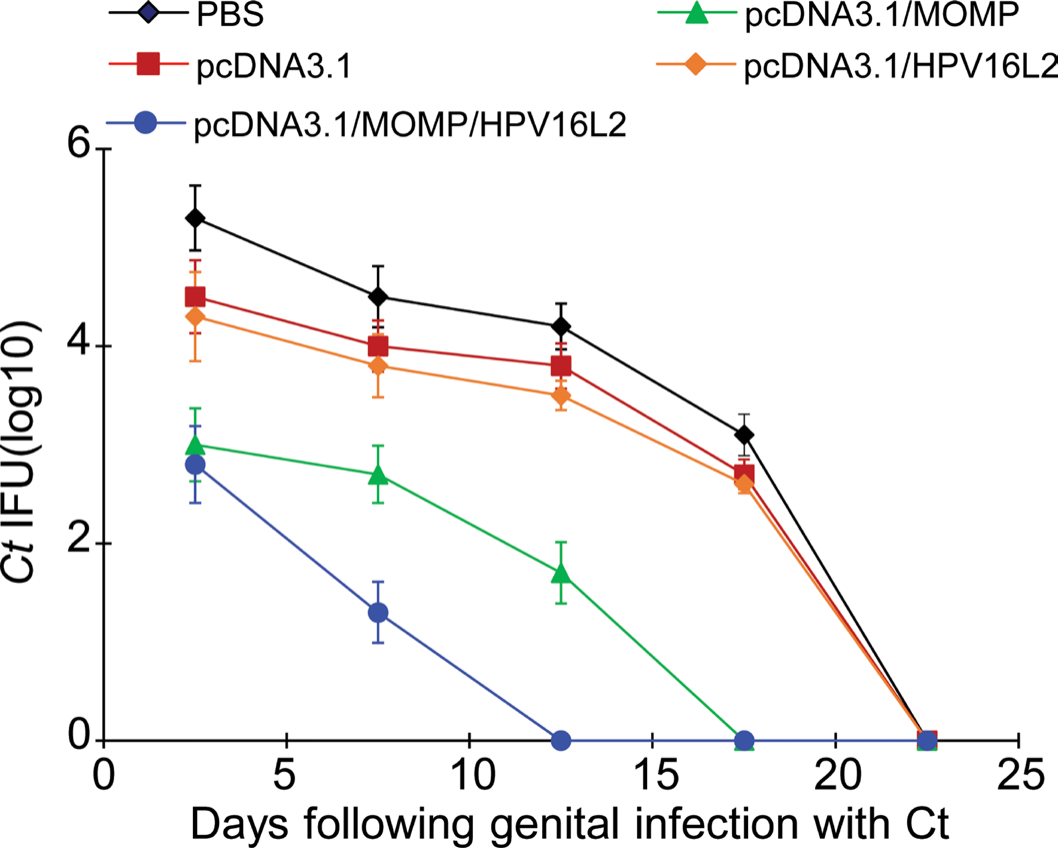
pcDNA3.1(+)/MOMP/HPV16L2 vaccine prevented *Ct* infection *Ct* shedding (inclusion-forming units, IFU) was estimated in vaginal fluids every 5 days post-challenge. The quantity of Ct released by mice administered with the pcDNA3.1/ MOMP/HPV16L2 chimeric plasmid was reduced significantly. Mice in the pcDNA3.1/MOMP/HPV16L2 group were Ct-positive for 15 days. In contrast, the pcDNA3.1/*Ct* MOMP multi-epitope group, pcDNA3.1/HPV16L2 group, pcDNA3.1 group and PBS groups showed Ct-positive cultures ranging between 20 and 25 days.

In this study, mice with different immunization were challenged intra-vaginally with 10^7^ inclusion forming units (IFU) *Ct* to test the protective immunity generated by the vaccines. To assess the protective effect from the immunization, *Ct* was extracted from the vaginal by using swabs and the IFUs in the vaccinated mice were compared at specific time points. We found an increased protection against *Ct* infection in mice immunized with pcDNA3.1/MOMP/HPV16L2 plasmid vaccine, based on the IFU number and infection clearance speed (Figure 8). Mice immunized with the pcDNA3.1/MOMP/HPV16L2 chimeric vaccine has cleared *Ct* on day 15, which was the fastest among all groups (Figure 8). Additionally, mice immunized with pcDNA3.1/MOMP/HPV16L2 plasmid vaccine had the lowest level of IFU, indicating the strongest protective through vaccination (Figure 8). The infection was completely cleared in all the animals treated with pcDNA3.1/MOMP/HPV16L2 within two weeks after initial challenge (Figure 8). The results suggest that a recombinant vaccine constructed on the HPV16L2 platform confers effective protection against Ct infection.

### pcDNA3.1(+)/Ct MOMP multi-epitope/HPV16L2 reduced mouse genital tract tissue pathologies

**Figure 9:**
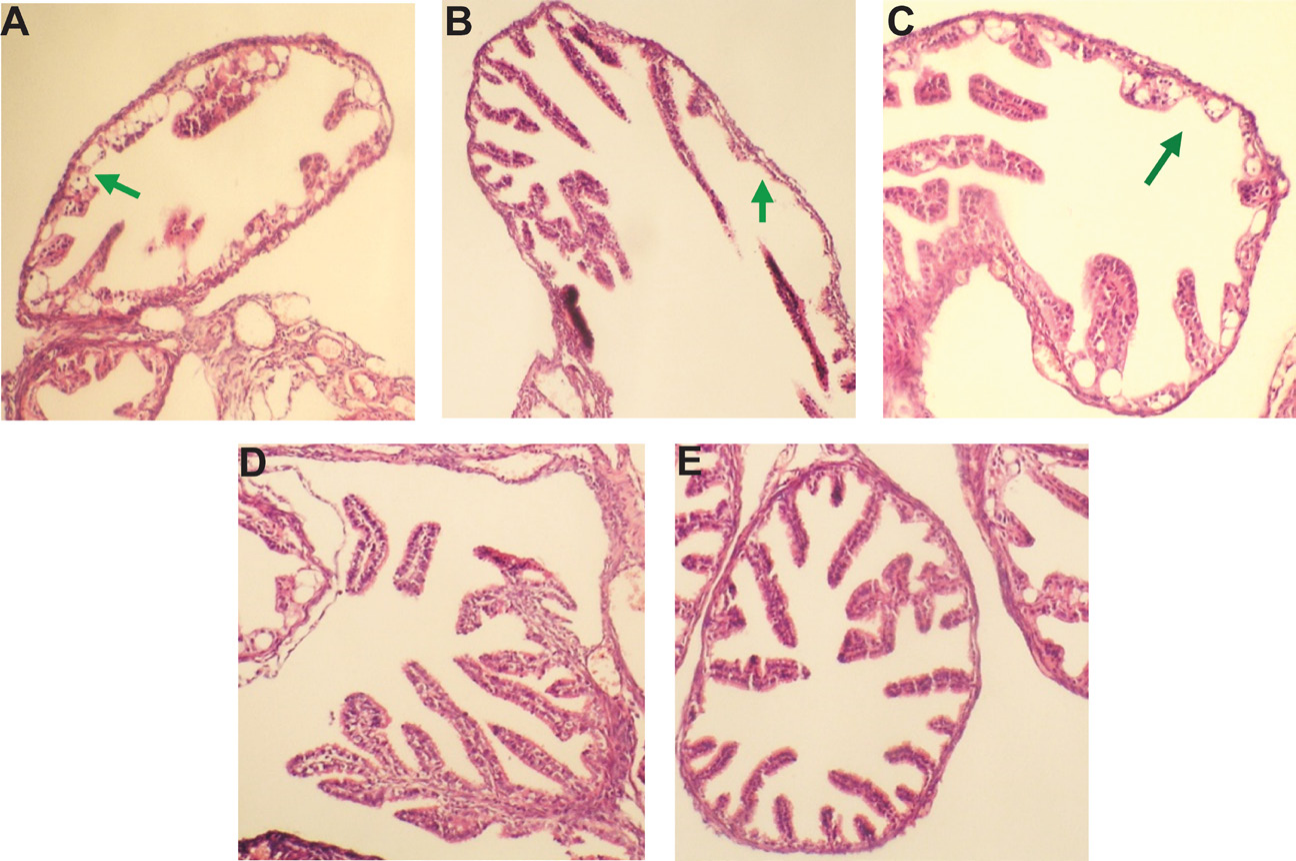
pcDNA3.1/Ct MOMP multi-epitope/HPV16L2 reduced mouse genital tract tissue pathologies Mice were infected with Ct. and treated with (**A**) PBS, (**B**) pcDNA3.1, (**C**) pcDNA3.1/HPV16L2, (**D**) pcDNA3.1/MOMP, and (**E**) pcDNA3.1/MOMP/HPV16L2. Mice received (D) pcDNA3.1/MOMP, and (E) pcDNA3.1/MOMP did not show observable pathological changes in the genital tract tissues. Mice treated with (A) PBS, (B) pcDNA3.1, and (C) pcDNA3.1/HPV16L2 showed tissue pathologies due to the infection, as indicated by the arrows.

The mice infected *Ct* had appeared different levels of hydrosalpinx and swelling, which indicated reproductive tract infection (Figure 9A). Similarly, immunization of *Ct*-infected mice with pcDNA3.1, pcDNA3.1/HPV16L2 did not eliminate hydrosalpinx and swelling, indicating these vaccination cannot completely protect the mice from reproductive tract damage from the infection (Figure 9A, 9B and 9C). Instead, in mice vaccinated with pcDNA3.1/MOMP or pcDNA3.1/MOMP/HPV16L2, no hydrosalpinx and swelling was oberserved, indicating an effective protection from the immunization (Figure 9D and 9E). Together, these studies illustrated the effectiveness of pcDNA3.1/MOMP/HPV16L2 in protecting *Ct* infected mice from reproductive tract damage – an important characteristic for the reproduction in the patient females.

## DISCUSSION

More than 100 million cases of reproductive tract infection by *Ct* occurs annually worldwide [1]. Despite decades of study, no *Ct* vaccine exists in the market yet. Studies show that MOMP represents the target antigen in *Ct* vaccine research because of the location of different epitopes [14]. In this study, the *Ct* MOMP multi-epitope (GDNENQSTVKTNSVPNMSLDQSVVELY TWQASLALSYRLNMFTPYIGV) that including 28-amino-acid (138–165) and 19-amino-acid sequences (249–268) was selected [6]. These amino acid sequences contain T helper cell (Th) epitopes, and epitopes related to HLA-A2-restricted cytotoxic T lymphocyte (CTLs) and specific B-cells. However, an issue this multi-epitope-based vaccine is that it can only induce minor immune responses [15]. Thus, HPV16L2 was selected to boost the immune response during Ct MOMP epitopes vaccination. Additionally, recent studies demonstrated that HPV16L2 immunization can generate broad cross-reactive and cross-neutralizing antibodies against HPV infections [16]. These studies illustrated that HPV16L2 components can serve as two functional roles in the vaccines: enhancing vaccination potency and generating antibodies against HPV infections, making this component an extremely attractive candidates for Ct vaccine design. In addition, HPV16, as the most risky HPV all over the world, are composed of L1 and L2 proteins. HPV16L1 and HPV16L2 are the major and minor proteins, respectively. In our previous study in murine model, we investigated the use of HPV6B L1 for Ct vaccine development, and the vaccine showed protective mucosal immunity against Ct infection [17]. However, one potential issue in using HPV L1 based vaccines is that two vaccines have been licensed in the market for HPV treatment. Due to the existence of these licenses, the translational of our L2 based vaccine may have some potential patent issues [18]. Importantly, despite L2 is a minor component of the viral capsid, it is necessary for papillomavirus infection. Current study found that L2 facilitate the infection process by binding to a secondary viral receptor, which helps the virus to escape from the endosomes and deliver the viral genome to the nucleus of the infected cells [19, 20]. In animal papillomavirus models, study suggested that the L2 component is a promising antigen for preventive vaccine, where the L2 peptide yielded effective protection against papillomavirus challenge at both mucosal and cutaneous sites [18, 19]. Other study also suggested that L2 can help improve the cancer vaccination efficacy compared to L1 based vaccine [21].

Studies also reported that without an appropriate immunogen, MOMP multi-epitope DNA alone evokes a less potent immune response than vaccines with immunogens [17], indicating that it is important to add an adjuvants to boost the immunity. To enhance the level, durability and extent of cross-protection against sexually transmitted infections (STIs), we designed a concatenated fusion protein consisting of *Ct* MOMP multi-epitope and HPV16L2. The addition of HPV16L2 into our vaccine is also supported by human trial study. For example, in one study, human vaccination with HPV16L2 yielded a very sensitive production of neutralizing serum antibodies that can be harnessed to protect patients from infection [22].

After immunization with pcDNA3.1/MOMP/HPV16L2 chimeric plasmid, specific immune response was detected in the serum of mice, indicating immunogenicity of *Ct* MOMP and HPV16L2 in the chimeric plasmid. The antibodies induced by *Ct* MOMP multi-epitope even reacted with the whole *Ct*. The IgA antibody was detected in the genital washes of immunized mice, indicating that the chimeric plasmid induced specific mucosal immunity locally. Additionally, CTL responses showed that the chimeric plasmid also induced cell-mediated immunity.

Compared with the *Ct* MOMP multi-epitope group, the *Ct* MOMP/HPV16L2 chimeric plasmid group induced higher anti-*Ct* IgG titer in serum and IgA titer in local mucosal secretions, and exhibited a significantly higher cytotoxicity (*p* < 0.05). Similarly, the *Ct* MOMP multi-epitope/HPV16L2 chimeric plasmid group induced higher anti-HPV16L2 IgG titer in serum and cytotoxicity compared with the HPV16L2 group, indicating greater immunogenicity of the fusion protein compared with the single *Ct* MOMP multi-epitope or HPV16L2 group. The study showed that the *Ct* MOMP/HPV16L2 chimeric plasmid group promoted elimination of *Ct*, resulting in reduced *Ct* colonization in the genital tract and significantly shorter *Ct* infection, indicating that the *Ct* MOMP/HPV16L2 chimeric plasmid played a protective role.

In conclusion, we report the successful design of a genetically engineered eukaryotic expression system composed of *Ct* MOMP/HPV16L2 gene. The recombinant DNA vaccine that contains *Ct* MOMP multi-epitope and HPV16L2 boosted the immune response to *Ct* infection *in vitro* and in mice, indicating the potentials of developing Ct vaccines.

## MATERIALS AND METHODS

### Materials

The pcDNA3.1 (+)/HPV16L2 and pcDNA3.1 (+)/*Ct* MOMP gene plasmids were designed in our lab, as reported before [17]. Restriction enzymes (i.e. *Xho*I, *Hind*III, and *EcoR*I) and T4 DNA Ligase were provided by Fermentas (Massachusetts, USA), and lipofectamineTM2000 kit was obtained from Invitrogen (Massachusetts, USA). Polyclonal rabbit IgG Abs against the HPV6bL2 and *Ct* were prepared in our lab, as reported before [17]. FITC-labeled goat anti-rabbit IgG, horseradish peroxidase (HRP)-labeled goat anti-mouse IgG and horseradish peroxidase (HRP)-labeled goat anti-rabbit IgG were obtained from Unitech (Chiba, Japan).

### Cells and animals

COS-7 cells were ordered from ATCC and cultured in our laboratory.COS-7 cells originated from African green monkey kidney and it is a fibroblast-like cell line suitable for vector transfection [17]. Female BALB/c mice (4 to 6 weeks old) were obtained from the Shanghai Experimental Animal Center. All animal experiments were compliant with the local and governmental regulations.

### Design of Ct MOMP multi-epitopes /HPV16L2 plasmid

Using PCR, the *Ct* MOMP multi-epitopes were amplified. The PCR primers includes: the forward primer (P1), 5′- CCCAAGCTTATGGGCGATAATGAA -3′ and the reverse primer (P2), 5′- CCGGAATTC GACTCCAATATATGGTGTA -3′. The pcDNA3.1(+)/HPV16L2 and *Ct* MOMP multi-epitope sequences were subjected to restriction endonuclease digestion with *Hind*III and *EcoR*I, and linked using T4 DNA Ligase to obtain the plasmids, pcDNA3.1(+)/*Ct* MOMP multi-epitope/ HPV16L2 (Figure 1). Successful design of the plasmids was confirmed using restriction endonuclease and sequencing analysis [17].

### Transfection

Lipofectamine TM2000 Kit was used to transfect COS-7 cells (60% to 80% confluent, around 10^6^ cells) with pcDNA3.1(+)/*Ct* MOMP multi-epitope/HPV16L2, pcDNA3.1(+)/*Ct* MOMP multi-epitope, and pcDNA3.1(+)/HPV16L2, respectively. The empty pcDNA3.1(+) vector served as a negative control. Six hours later, DMEM supplemented with 10% fetal bovine serum (Gibco, USA) was used. The gene and protein expression were evaluated two days after transfection.

### RT-PCR

RNA was extracted from the COS-7 cells using TRIzol. 2 μg RNA was reverse transcribed into cDNA to serve as a template for the PCR amplification of HPV16L2 (1456 bp) and Ct MOMP multi-epitope (159 bp). Primers for HPV16L2 amplification were used: forward (P3), 5′- GGAATTCATGCGACACAAACGTTCTGCAAA ACGCACAAAACG -3′ and reverse (P4), 5′- CCGCTCG AGTTAGTGGTGGTGGTGGTGGTGGGCAGCCAAA GAGACAT -3′. The RT-PCR products were verified by electrophoresis on a 2% agarose gel.

### Confocal immunofluorescence microscopy

For confocal microscopy imaging, COS-7 cells were transfected with the plasmids for 2 days. After the transfection, the cells were then treated with PBS containing 0.5% Tween 20 (PBS-T) three times for 10 min. The cells were then fixed with 1% paraformaldehyde (Sigma) supplemented with 0.1% Triton X-100 (Sigma). Following fixation, the cells were treated with polyclonal anti-*Ct* Ab and polyclonal anti-HPV16L2 Ab (1:200), respectively, and then incubated with FITC-conjugated goat anti-rabbit Ab (1:1000) for staining. The cells were subjected to 0.1 μg/ml of propidium iodide (PI) for 10 min to stain the nuclei. The stained cells were visualized under a Leica TCS SP2 microscope on glass slides (Germany).

### Western blotting

2 days after transfection, proteins in the COS-7 cells were exposed to M-PER Mammalian Protein Extraction Reagent Kit. The proteins were subjected to 10% SDS-PAGE gel and transferred to a PVDF membrane electrophoretically, followed by soaking with 5% non-fat milk. The proteins were incubated separately with polyclonal rabbit anti-HPV16L2 (1:200) and anti-*Ct*(1:200) for 1 h, and subsequently with an HRP-coupled goat anti-rabbit Ab (1:5000). Inhibition, incubation, and washing were carried out in TBST (Tris-Buffer Saline with Tween 20). Proteins were quantified visually using the DAB kit (Pierce Co, Thermo Fisher Sci).

### Immunization protocol

The DNA vectors were injected into female BALB/c mice intramuscularly at intervals of 1, 3, 5 and 7 weeks. Five different groups of mice (12 each) were included: group 1 was injected with PBS (100 μl); group 2 was administered with 100 μg pcDNA 3.1(+) in 100 μl PBS; group 3 was given 100 μg pcDNA3.1(+)/*Ct* MOMP in 100 μl of PBS; group 4 was treated with 100 μg pcDNA3.1(+)/HPV16L2 in 100 μl PBS; and group 5 with 100 μg pcDNA3.1(+)/*Ct* MOMP multi-epitope /HPV16L2 in 100 μl PBS.

### ELISA

An alkaline phosphatase-based ELISA was used to quantify serum Ct-specific IgG and secretory IgA in the vaginal washes. The amounts of HPV16L2-specific IgG in serum were quantified using an alkaline phosphatase-based ELISA. ELISA plates (Nunc, Demark) were used to incubate 10 μg/ml inactivated Ct serovar E(EB) and HPV16L2 protein in 100 μl carbonate buffer (pH 9.6) overnight. After washing three times with PBS-T, the plates were blocked with 2% BSA in PBS. The well contained either 100 μl serum or 50 μl vaginal wash (1:100) in triplicate. The plates were incubated at 37°C for 90 min. After washing with PBS-T, 100 μl HRP-conjugated goat anti-mouse IgA or IgG (1:5000) was added to each well for 1 h at 37°C. Upon adding orthophenylenediamine (OPD) to the plates, the absorbance was at 490 nm read immediately.

### Cytotoxic T-lymphocyte (CTL) assay

Splenocytes were isolated from the mice in 1 week after the final immunization. Splenocytes (1 × 10^7^) and inactivated EB (2 × 10^5^) were incubated with 0.5 ng/ml IL-7 and 20 U/ml IL-2 in 24-well tissue culture plates. On day 5 of culture, the viable splenocytes were used as effector cells. A lactate dehydrogenase release assay (Roche) was used to determine the cytotoxicity of the cultures. The P815 cells were pre-incubated with CTL peptide 176-185 MSLDQSVVEL (10 μg/ml) from *Ct* MOMP multi-epitope for 4 h, washed in RPMI 1640 containing 5% FBS, and targeted by the stimulated splenocytes. Untreated P815 cells were used as the target cell controls. The stimulated splenocytes along with target cells were incubated at different effector-target cell (E/T) ratios of 20:1, 10:1, and 5:1 in 96-well round-bottom culture plates at 37°C in 5% CO_2_, followed by centrifugation at 1500 rpm for 10 min, twice, 4 h apart. 50 μl supernatant were added to another plate supplemented with the substrate cocktail (50 μl/well), and stopped after 30 min of incubation in the dark to record the optical density at 490 nm. Specific lysis was calculated as follows: % specific lysis = 100 × [(release in the presence of CTL−spontaneous release)/(maximal release−spontaneous release)][17, 23]. In all the experiments, the spontaneous release was less than 30% of the maximal release.

### Protection studies

The mice in groups of 12 were immunized as described above. Three weeks after the final immunization, the mice were challenged intravaginally with 10^6^ inclusion-forming units of live *Ct* serovar E. Infection was determined by isolating the *Ct* from cervical-vaginal swabs. *Ct* was isolated by inoculating HeLa cell cultures using centrifugation as described above.

### Evaluating mouse genital tract tissue pathologies

Mice were sacrificed 12 days after infection, and the genital tract tissues were isolated. The tissues were fixed in formalin, embedded in paraffin, and sectioned 5 μm/each section. The sections were stained with hematoxylin and eosin (H&E) and were assessed by a pathologist blinded to mouse treatment [23].

### Statistical analysis

Student's *t* test was used to analyze the levels of secretory IgA, IgG and protection. A probability value less than 0.05 was deemed significant.
